# Supplementary material for: The Proteomics of T-Cell and Early T-Cell Precursor (ETP) Acute Lymphocytic Leukemia: Prognostic Patterns in Adult and Pediatric-ETP ALL
Source: Cancers (Basel). 2024 Dec 19;16(24):4241. doi: 10.3390/cancers16244241 (PMC11674289; doi:10.3390/cancers16244241)
Supplement: Supplementary file 1 [file cancers-16-04241-s001.zip › cancers-3284066-SM-2-done v3.pdf]

cancers MDPI

Table S1. Treatment regimens T-ALL patients (N=361).

Regimen	Number
AFBM*	139
AFBM + bortezomib*	150
Ineligible*	3
AUG BFM	10
HyperCVAD (HCVAD)	23
Augmented-HCVAD	3
HCVAD + Nelarabine	28
Mini-HCVD + Venetoclax	1
VAD	1
CLIA1 + sorafenib 1	1
Clofarabine + etoposide +cyclophosphamide + vincristine + bortezomib	1
Nelarabine	1

*All of these were treated on COG AALL1231.

Table S2. Protein allocation in the 32 protein functional groups.

Protein Functional Group	Protein
Adhesion	ANXA1, ANXA7, CDH1, CDH2, CD44, COL6A1, ITGAL, ITGA2, ITGB1, MMP2, MUC1, PECAM1, SPP1
Apoptosis Occurring	CASP3, CASP3.cle, CASP7.cle, CASP9, PARP1.cle
Apoptosis Regulating	AIFM1, BIRC2, CSNK2A1 XIAP
Apoptosis BH3	BAX, BAK1, BBC3, BCL2, BCL2A1, BCL2L1, BCL2L11, BID, MCL1
Autophagy	ATG3, ATG7, BECN1, PRKAA1_2, PRKAA1_2.pT172, SQSTM1, STK11
Cell Cycle	CCNB1, CCND3, CCNE1, CCND1, CDC25C, CDKN1A, CDKN1B, CDKN1B.pS10, CDKN1B.pT157, CDKN1B.pT198, CDKN2A, CDK1, CDK2, E2F1, FOXM1, FOXO3A, FOXO3A.pS318_321, FZR1, PCNA, PLK1, RB1, RB1.pS807_811, WEE1
Checkpoint	CD276, CD86, PDCD1, PDL1, VTCN1
Creb	ATF3, CREB1, CREB1.pS133, EP300
Cytoskeletal	ACTB, COL6A1, MYH11, NOL3, STMN1, TUBA4A, VIM, XPO1
Differentiation	GATA1, GATA3. PIM1, PIM2, SOX2
DNA damage	ATM, ATM.pS1981, BRCA2, CHEK1, CHEK1.pS345, CHEK2, CHEK2.pT68, ERCC1, ERCC4, ERCC5, MSH2, MSH6, RAD50, RAD51, RPA32, RPA32.pS4_8, SSBP2, XPA, XRCC1
Heatshock	GRP78, HSF1, HSF1.pS326, HSPA1A_L, HSPB1, HSPB1.pS82, HSP90AA1_B1
Hippo	DVL3, LATS1, NF2, STK11, STK4, TAZ, YES1
Histone modification	ASH2L, BRD4, CLPP, EZH2, HDAC1, HDAC2, HDAC3, HDAC6, HIST3H3, HNRNPK, H2BFM, H3K4Me1, H3K4Me2, H3K4Me3, H3K9Me2, H3K27Me3, H3K36Me3, KDM1A, KMT2A, KMT2B, MEN1, NCL, NPM1, SETD1A, SETD1B, SIRT1, SIRT6, SUZ12, WDR5, WDR82, WTAP
Hypoxia	EGLN1, EP300, HIF1A, KDR, VHL
MAPK	MAPK1, MAPK1_3.pT202_204, MAPK8_10, MAPK8_10.pT183_185, MAPK9, MAPK14, MAPK14.pT180_182, MAP2K1, MAP2K1.pS217_221, MAP2K2, MKNK1

Mek	ARAF, BRAF, BRAF.pS445, MET.pY1230_1234_1235, NRAS, RAB25, RAF1, RAF1.pS338, RHEB
Metabolic	ACACA, ACACA_B.pS79, ASNS, ASS1, FASN, GAPDH, GLS, GPBB, GSK3A_B, GSK3A_B.pS21_9, GYS1, GYS1.pS641, NDUFB4, NLN, PKM2, PPARA, PPARG, PRKAA1_2, PRKAA1_2.pT172, PTGS2, SCD, SDHA, TGM2
mTOR pathway	AKT1S1, AKT1S1.pT246, MTOR, PDK1, PDK1.pS241, RICTOR, RICTOR.pT1135, RPTOR, TSC1, TSC2, TSC2.pT1462
Phosphatase	DUSP4, PPP2RA_B_C_D
PIK3AKT	AKT1, AKT1_2_3, AKT1_2_3.pS473, AKT1_2_3.pT308, AKT2, AKT3, FOXM1, FOXO3A, FOXO3A.pS318_321, GSK3A_B, GSK3A_B.pS21_9, INPPL1, PIK3CA, PIK3CB, PRKAR1A, PTEN
PKC	PEA15, PRKCA, PRKCA.pS657, PRKCB, PRKCB.pS660, PRKCD.pS664
Ribosome	ADAR, DDX17, EEF2, EEF2K, EIF2AK2, EIF2AK2.pT451, EIF2S1, EIF2S1.pS51, EIF4E, EIF4EBP1, EIF4EBP1.pT37_46, EIF4G1, EIF4G2, HNRNPK, NDRG1.pT346, PDCD4, RPSKA1.pT573, RPS6, RPS6.pS235_236, RPSKA1_2_3, RPS6KB1, RPS6KB1.pT389, SRSF1, TFRC
Smad	SMAD1, SMAD2, SMAD2.pS245_250_255, SMAD2.pS465_467, SMAD3, SMAD4, SMAD5, SMAD5.pS463_465
Src	LCK, LYN, SRC, SRC.pT527, YES1
STAT	JAK2, STAT1, STAT3, STAT3.pS727, STAT3.pT705, STAT5A
STP	AXL, EGFR, EGFR.pT1173, ERBB2, ERBB2.pT1248, GAB2, GAB2.pT452, IGFBP2, IGF1R, IGF1R.pY1135_1136, KIT, LYN, PDGFRB, PREX1, PTK2, PTK2.pT397, PTPN11, PTPN11.pT542, SHC1.pT317, SYK, TYRO3,
T-cell	CD74, CD86, JAG1, LCK, NOTCH1, NOTCH1.cle, NOTCH2, NOTCH3, TAPBP
TP53	ATM, ATM.pS1981, MDM2, MDM2.pS166, MDM4, NUMB, TP53, TP53.pS166
Transcription	ARID1A, FLI1, ELK1.pS383, ERG, ETS1, HES1, JUN.pS73, MEF2C, MYC, RELA, RELA.pS536, SPI1, YBX1.pS102
Unfolded protein response	CAV1, COPS5, EIF2S1, EIF2S1.pS51, ERN1, GRP78, VCP
Wnt signaling	CTNNB1, CTNNB1.pS33_37_T41, CTNNB1.pT41_S45, NCSTN

Table S3. Constellation membership for each protein cluster (N=125).

CON	Protein Cluster
1	adhesion C4
1	checkpoint C4
1	histone C4
1	hypoxia C3
1	mapk C4
1	mek C3
1	metabolic C3
1	phosphatase C4
1	pi3kakt C4
1	smad C4
1	stp C5
1	tp53 C4
1	upr C4
2	apopoccur C2
2	bh3 C4
2	cellcycle C3

2	creb C3
2	differentiation C3
2	dna damage C3
2	heatshock C2
2	mtor C2
2	pkc C1
2	ribosome C5
2	stat C3
2	transcription C2
2	wnt C4
3	apopreg C3
3	cytoskeletal C2
3	hippo C1
3	hypoxia C1
3	mapk C2
3	phosphatase C3
3	src C1
3	tcell C1
3	tcell C2
3	wnt C5
4	checkpoint C2
4	mek C2
4	metabolic C5
4	pi3kakt C5
4	smad C2
4	stp C4
4	tp53 C5
4	upr C2
5	dna damage C2
5	histone C2
5	mtor C3
5	stp C1
6	creb C1
6	differentiation C2
6	dna damage C1
6	hypoxia C2
6	mek C1
6	mtor C1
6	pi3kakt C1
6	stp C3
6	tcell C3
6	transcription C3
6	upr C1
7	adhesion C3
7	cellcycle C2
7	hippo C2
7	src C2
7	stat C2
7	transcription C1
8	apopoccur C1
8	checkpoint C1

8	creb C2
8	phosphatase C1
8	smad C3
8	tp53 C2
9	apopreg C2
9	autophagy C2
9	checkpoint C3
9	cytoskeletal C3
9	heatshock C4
9	histone C3
9	metabolic C2
9	ribosome C4
9	wnt C1
10	adhesion C2
10	apopoccur C3
10	apopreg C1
10	autophagy C1
10	bh3 C1
10	cellcycle C1
10	cytoskeletal C1
10	heatshock C5
10	histone C1
10	mapk C1
10	metabolic C1
10	ribosome C1
10	smad C1
11	autophagy C3
11	heatshock C3
11	hippo C3
11	hypoxia C4
11	mek C4
11	metabolic C4
11	pi3kakt C3
11	ribosome C2
11	src C3
11	stat C5
12	adhesion C1
12	autophagy C4
12	bh3 C2
12	heatshock C1
12	mapk C3
12	pkc C2
12	pkc C3
12	ribosome C3
12	stat C4
12	wnt C2
13	bh3 C3
13	differentiation C1
13	phosphatase C2
13	pi3kakt C2
13	pkc C4

13	pkc C5
13	stat C1
13	stp C2
13	tp53 C1
13	tp53 C3
13	upr C3
13	wnt C3

Table S4. Pediatric-dominant (ETP-P; pink) and adult/pediatric mixed samples (ETP-MX; green).

	Type	Count	Freq	ETP-P	ETP-MX	*P*
**Cluster**	Count	64	100%	23%	77%	
**Gender**	Female	18	28%	40%	24%	0.400
	Male	46	72%	60%	76%	
**Ethnicity**	Hispanic or Latino	14	24%	23%	24%	1.000
	Not Hispanic or Latino	45	76%	77%	76%	
**CNS-involvement**	Yes	14	22%	7%	27%	0.204
	No	50	78%	93%	73%	
**Age (continuous)**	Mean	11.8		12.2	11.7	0.699
	SD	4.6		4.4	4.6	
**Infant**	Yes	1	2%	0%	2%	
	No	56	98%	100%	98%	1.000
**MRD (end of induction II)**	Yes	42	66%	67%	65%	
	Neg	15	23%	20%	24%	
	Unknown	7	11%	13%	10%	1.000
**MLL-rearrangement**	Yes	7	11%	13%	10%	
	No	51	80%	73%	82%	1.000
	NA	6	9%	13%	8%	
**ETP type**	Positive	24	38%	53%	33%	0.253
	Near	40	63%	47%	67%	
**Risk stratification (AALL1231)**	Standard risk	13	20%	20%	20%	
	Intermediate risk	36	56%	53%	57%	0.985
	Very high risk	8	13%	13%	12%	
	NA	7	11%	13%	10%	
**WBC (continuous)**	Mean	166.6		47.9	202.9	0.007
	SD	199.3		52.7	213.5	

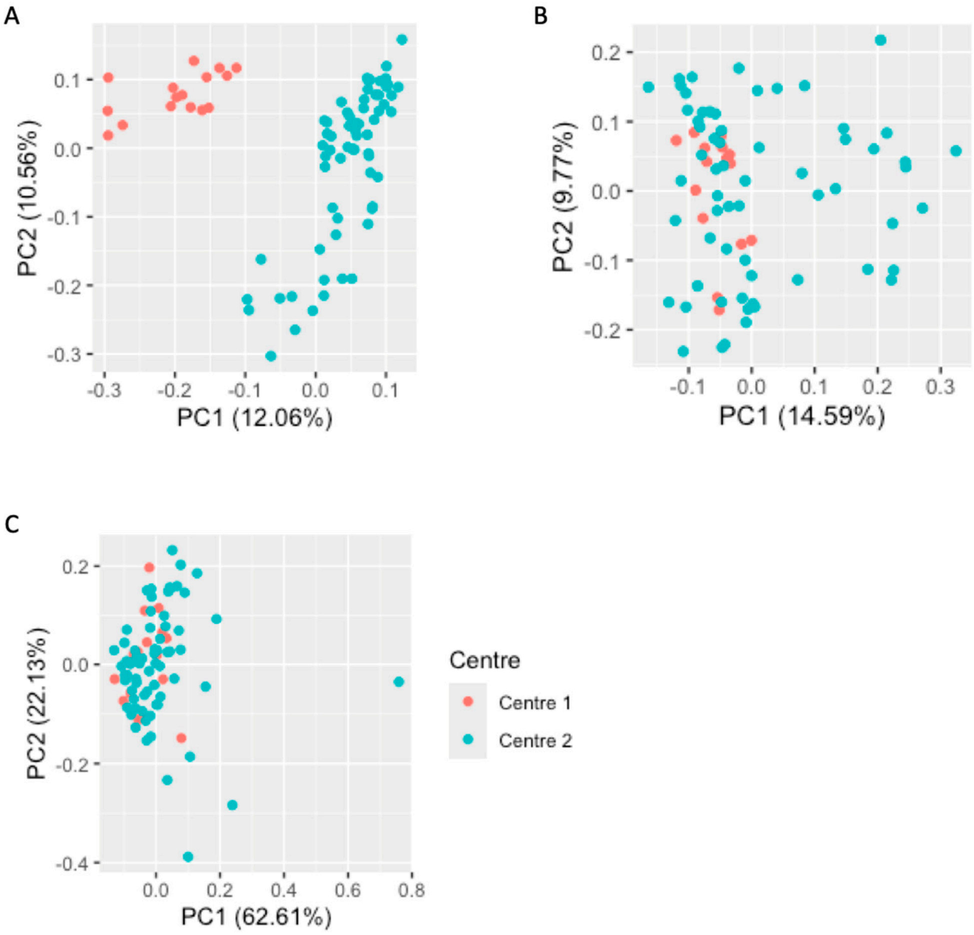

Figure S1. Principal component analysis (PCA) of RNA sequencing data across two batches A. before normalization B. after variance stabilizing transformation (VST) using DESeq2 and C. after batch correction with COMBAT.

A

	% ADULT SAMPLES				
	PC-1	PC-2	PC-3	PC-4	PC-5
adhesion	33%	28%	27%	6%	
apopoccur	35%	15%	22%		
apopreg	32%	16%	29%		
autophagy	30%	22%	44%	7%	
bh3	38%	29%	19%	6%	
cellcycle	43%	23%	10%		
checkpoint	35%	22%	25%	11%	
creb	29%	31%	16%		
cytoskeletal	34%	34%	8%		
differentiation	56%	25%	13%		
dna_damage	34%	24%	9%		
heatshock	19%	15%	50%	19%	47%
hippo	20%	20%	64%		
histone	41%	26%	17%	9%	
hypoxia	17%	31%	12%	49%	
mapk	37%	26%	18%	3%	
mek	25%	21%	9%	44%	
metabolic	34%	14%	10%	40%	31%
mtor	27%	10%	40%		
phosphatase	35%	20%	28%	9%	
pi3kakt	24%	41%	54%	8%	9%
pkc	13%	24%	40%	18%	38%
ribosome	55%	54%	10%	16%	11%
smad	40%	26%	23%	8%	
src	12%	28%	79%		
stat	37%	25%	25%	24%	14%
stp	40%	44%	26%	8%	9%
tcell	41%	23%	21%		
tp53	25%	26%	46%	15%	24%
transcription	29%	13%	29%		
upr	26%	22%	37%	16%	
wnt	23%	25%	50%	14%	21%

B

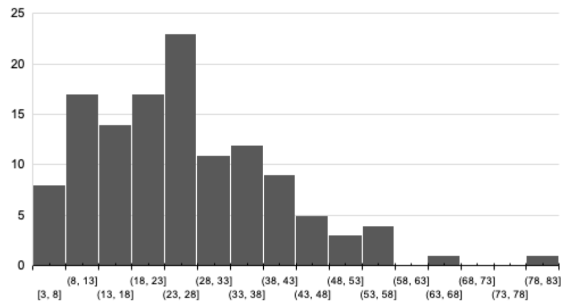

Median	25.00
Mean	26.12
Standard deviation (SD)	13.67
Median + 1 SD	38.67
Median + 2 SD	52.35
Median - 1 SD	11.33
Median - 2 SD	-2.35
Samples within +/- 1 SD	N=20/125 (16%)
Samples within +/- 2 SD	N=125/125 (100%)

Figure S2. Number of identified protein clusters (PC). A. Number of identified PC for each protein functional group (PFG). Percentages reflect the percentage (%) of adult samples within each PC. B. Distribution of the percentage of adult samples across all PC.

A B

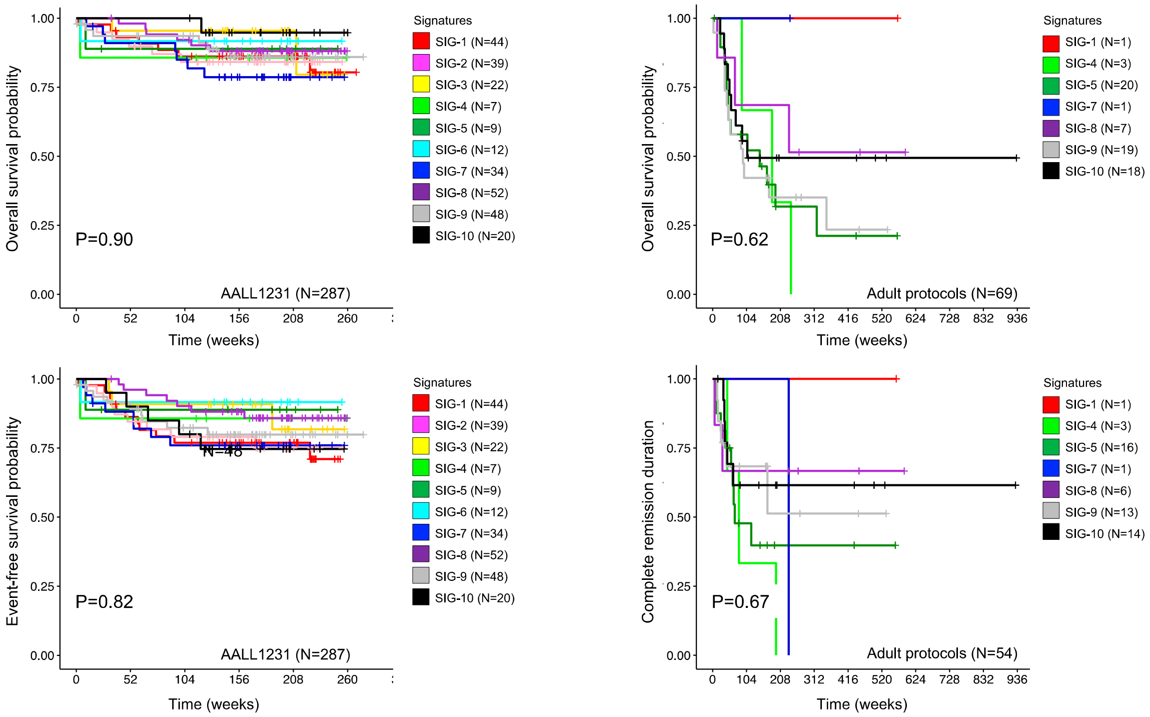

Figure S3. Kaplan-Meier Survival analysis for the 10 SIG. A. Overall survival (OS) (upper) and event-free survival (EFS) (lower) stratified by the 10 SIG for patients treated on the AALL1231 COG protocol. B. OS (upper) and complete remission (CR) duration (lower) for T-ALL patients treated on a variety of adult treatment protocols. Colors of each SIG correspond to annotation bar on top of the MG (Figure 1). SIGs were left out in the curves and the figure legend if they did not include any patients.

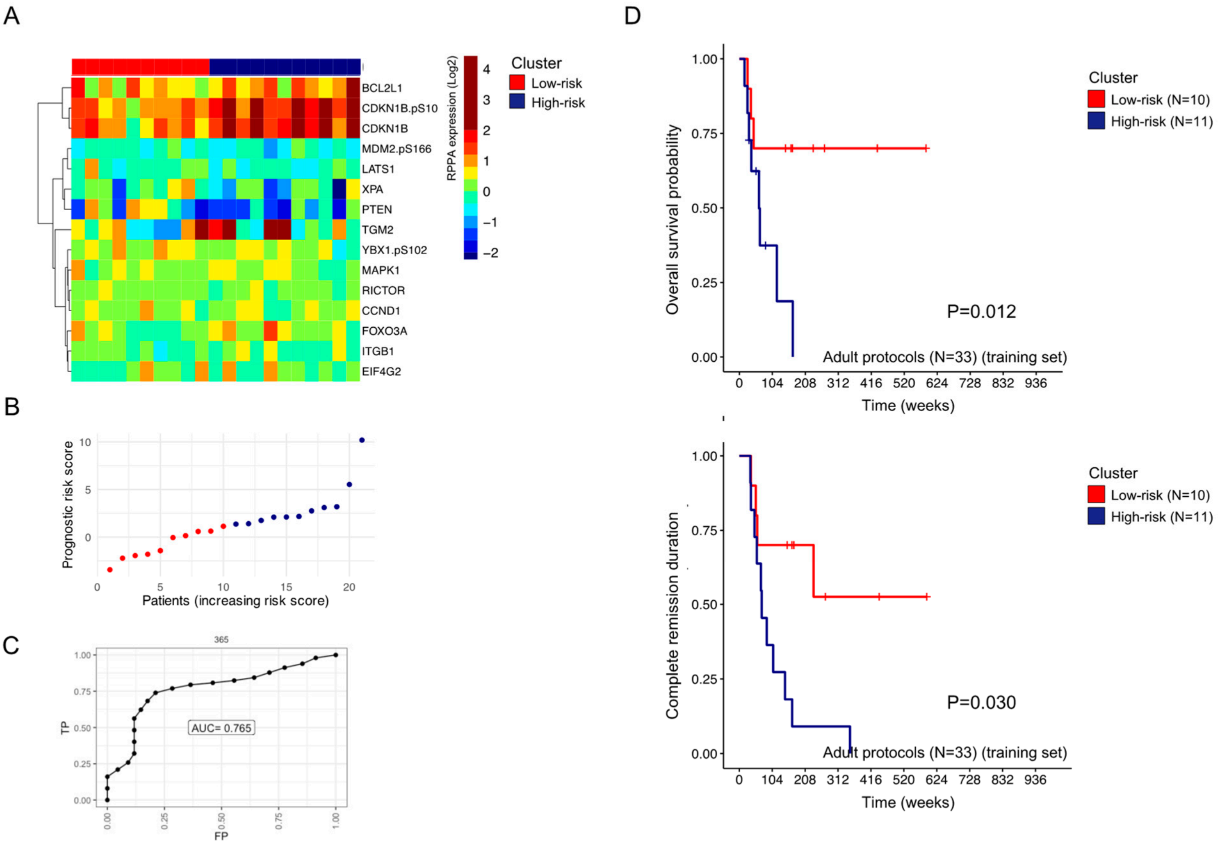

Figure S4. Regression analysis identified 2 protein clusters associated with clinical outcome in adult T-ALL in the test set (N=21). A. Heatmap showing relative protein expression levels of 15 proteins significantly associated with complete remission (CR) duration in the test set (N=21). B. Prognostic risk score calculated for each individual patient. Patients were divided into a low-risk and high-risk group based on the median risk score. C. Time-dependent receiver operator characteristic (ROC) analysis. D. Overall survival and CR duration stratified for patients in the low-risk (red) and the high-risk (navy blue) protein cluster.

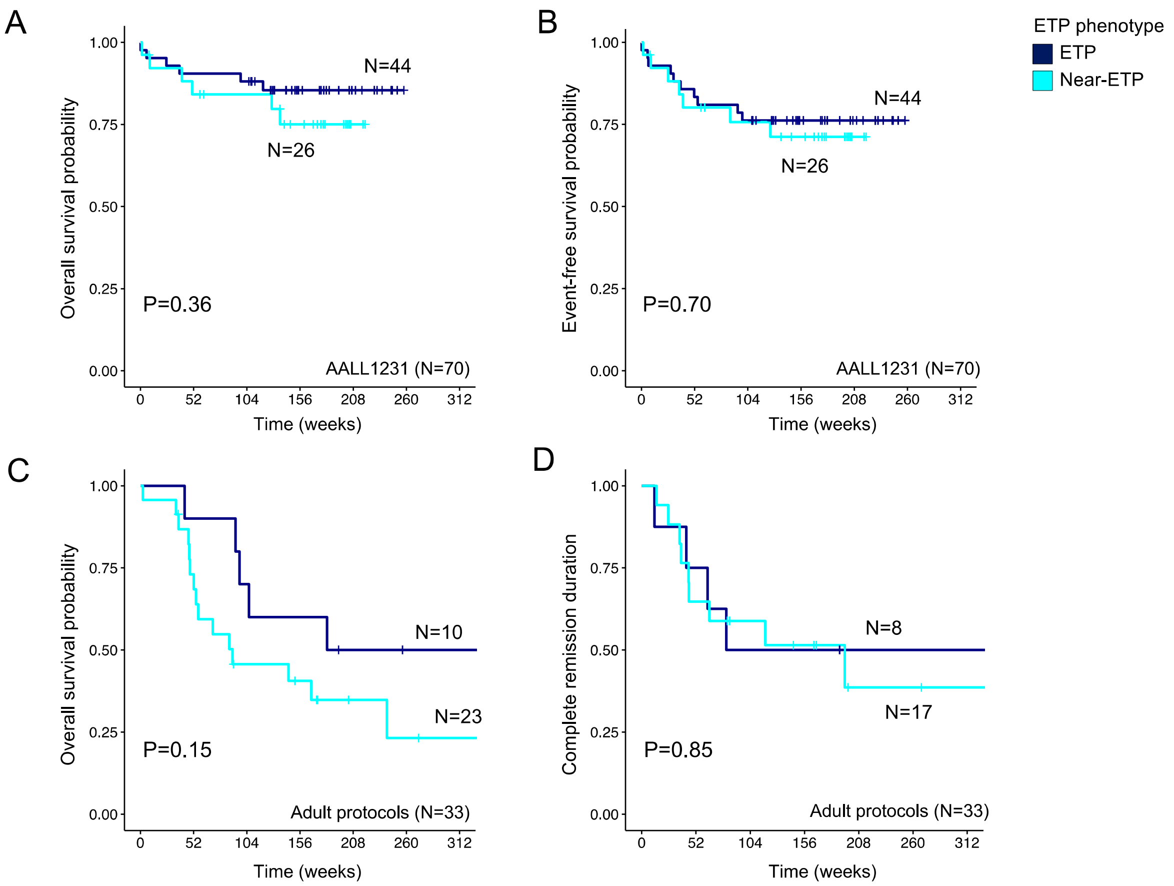

Figure S5. ETP and near ETP phenotype have similar outcomes in both adult and pediatric T-ALL. A. Overall survival (OS) (left) and B. event-free survival (right) for pediatric patients treated under AALL1231 stratified by ETP phenotype. C. OS and D. complete remission duration for patients treated on a variety of adult T-ALL protocols stratified by ETP phenotype.

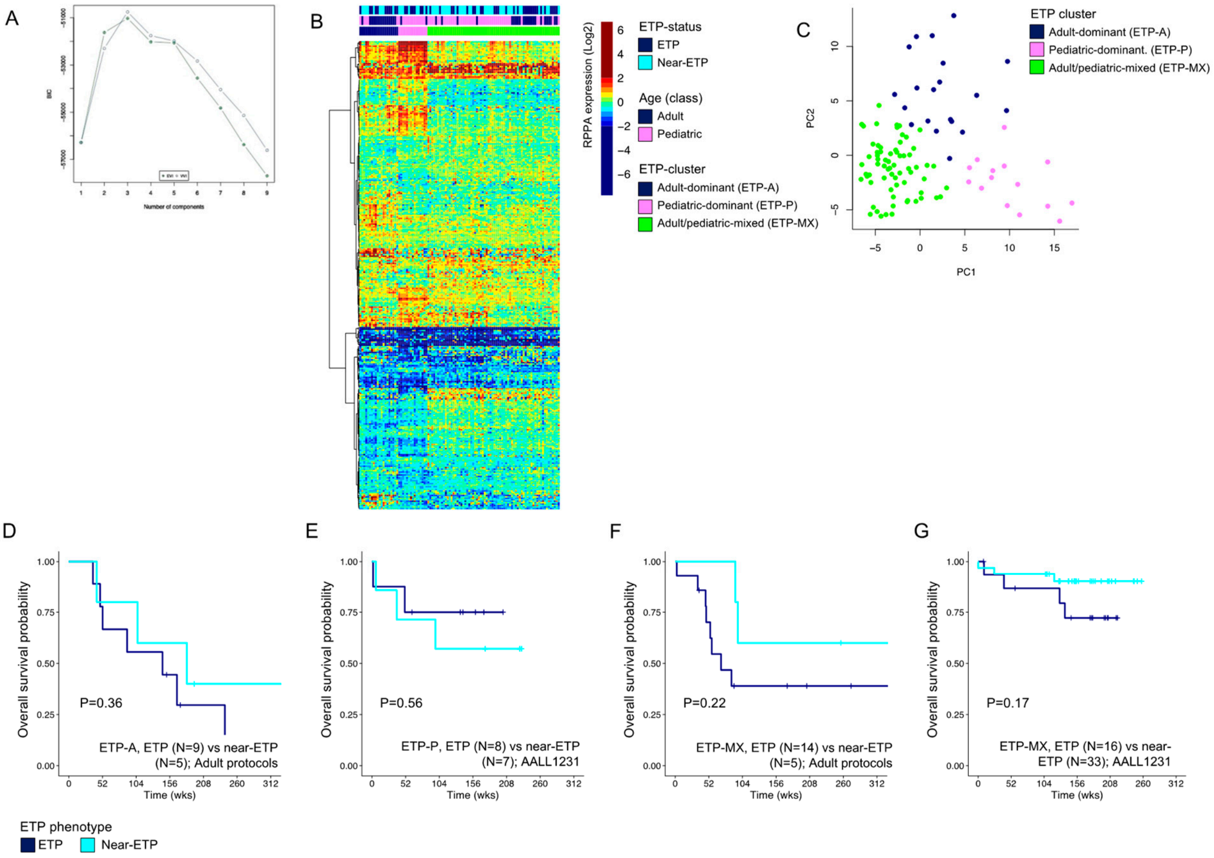

Figure S6. ETP-phenotype compared to near ETP T-ALL. A. Bayesian information criterion (BIC) score as a function of the number of components, with the optimal number of clusters determined as three. **B.** Unsupervised clustering heatmap showing the relative protein expression levels in pediatric and adult patients with ETP or near-ETP T-ALL. Annotations are shown for ETP-status: near-ETP (light blue) and ETP (navy blue); age (class): adults (navy blue), pediatric (pink). **C.** Principal component analysis of the three clusters. **D-G.** Overall survival stratified by ETP vs near-ETP phenotype for the three protein clusters: D, Adult-dominant (ETP-A); E, pediatric-dominant (ETP-P), F; mixed adult/pediatric (ETP-MX) treated on a variety of adult treatment protocol, and G; mixed adult/pediatric treated (ETP-MX) on COG AALL1231.

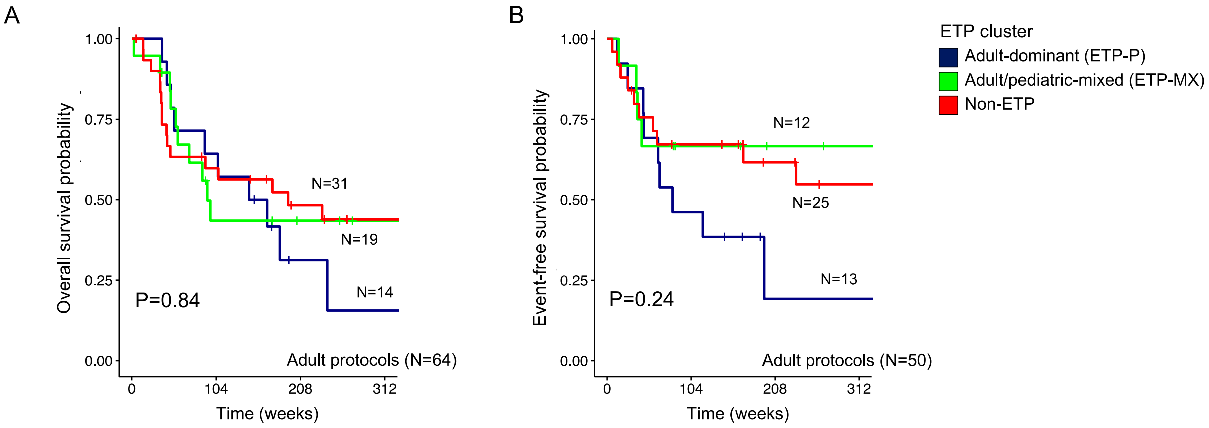

Figure S7. A. Overall survival and B. complete remission duration for the adult patients treated on a variety of adult T-ALL treatment protocols. Legend: blue: adult-dominant (ETP-A), green: adult/pediatric mixed protein clusters (ETP-MX). Non-ETP patients (red) were shown as a reference.

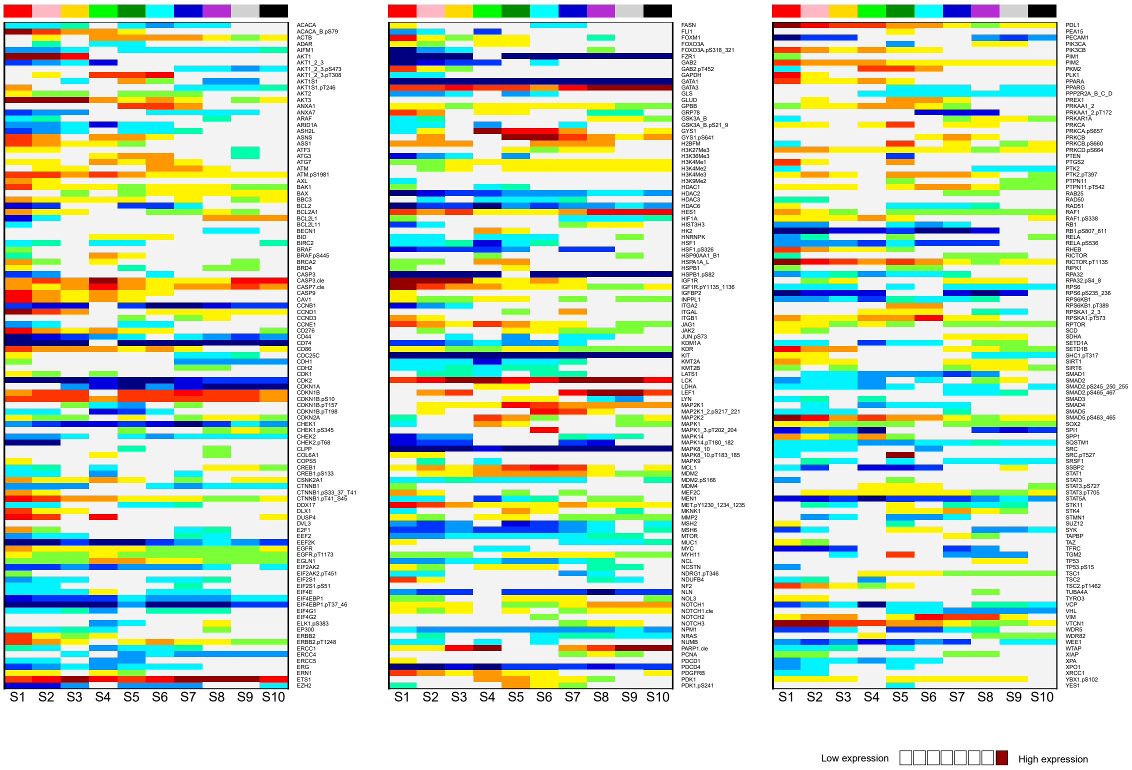

Figure S8. Median relative protein expression across the 10 identified protein expression signatures relative to the expression in the normal CD34+ cells. Proteins that were not significantly different from normal are shown in blank.
